# Extra-adrenal adrenocortical cancer associated with multiple endocrine neoplasia type 1

**DOI:** 10.1530/EDM-23-0068

**Published:** 2024-11-25

**Authors:** Deirdre Green, Kate Richards, Brendan Doyle, Chris Thompson, Arnold Hill, Michael W O’Reilly, Mark Sherlock

**Affiliations:** 1 Department of Endocrinology, Beaumont Hospital, Dublin, Ireland; 2 Academic Department of Endocrinology, Royal College of Surgeons in Ireland, Dublin, Ireland; 3 Department of Histopathology, Beaumont Hospital, Dublin, Ireland; 4 Department of Surgery, Royal College of Surgeons in Ireland and Beaumont Hospital, Dublin, Ireland

**Keywords:** Adrenal, Endocrine cancers, Please select country of treatment for each patient reported, IRELAND

## Abstract

**Summary:**

Adrenocortical carcinoma (ACC) is a rare malignant tumour arising from the adrenal cortex, with an estimated annual incidence of one to two patients per million. Ectopic ACCs are extremely rare. The majority of ACCs are sporadic; however, ACC has been linked with genetic disease processes, including multiple endocrine neoplasia type-1 (MEN-1). We present the case of a 66-year-old lady referred with newly diagnosed diabetes on a background of primary hyperparathyroidism. Examination revealed Cushingoid features, and hormonal evaluation confirmed ACTH-independent Cushing’s syndrome. Morning cortisol after a 1 mg overnight dexamethasone suppression test was 548 nmol/L with an undetectable ACTH <3.0 pg/mL. Dehydroepiandrosterone sulphate was 5.3 μmol/L and androstenedione 3.49 nmol/L, both of which were normal. Testosterone was suppressed at <0.4 nmol/L. Imaging revealed a 6 × 6 × 4.5 cm right-sided presumed adrenal lesion, a pancreatic lesion (2.5 × 1.6 cm), and bilateral pulmonary nodules (0.9 × 0.8 cm, 0.7 × 0.6 cm, 0.3 cm). Right adrenalectomy was performed, and histology was consistent with an extra-adrenal ACC (Weiss score 5/9) within the peri-adrenal adipose tissue. The resected adrenal gland was normal. Lung biopsy confirmed metastatic ACC tissue, and endoscopic ultrasound-guided biopsy of the pancreatic lesion revealed a pancreatic neuroendocrine tumour, which was confirmed biochemically to be an insulinoma. Genetic assessment confirmed MEN-1. This case highlights the importance of screening for MEN-1 in at-risk patients and the need for close clinical follow-up. To our knowledge, this is the first case report of extra-adrenal ACC in MEN-1 syndrome.

**Learning points:**

- Adrenal lesions in MEN-1 syndrome have significant malignant potential. Newly diagnosed lesions should be followed closely with short-interval imaging, and a lower threshold for surgical removal is suggested.
- Primary hyperparathyroidism is often the earliest laboratory or clinical manifestation of MEN-1 syndrome. A detailed medical and family history is vital in order to appropriately identify patients at risk of MEN-1.
- To our knowledge, this is the first case report of extra-adrenal adrenocortical carcinoma in MEN-1 syndrome.

## Background

Adrenocortical carcinoma (ACC) is a malignant tumour arising from the adrenal gland, specifically the adrenal cortex. ACCs are rare, with an estimated annual incidence of one to two patients per million (1). ACC is aggressive with an overall poor prognosis. Survival can be heterogeneous depending on the extent of the disease; however, 5-year survival rates of only 10–15% are reported in metastatic disease (1). Approximately 50% of lesions are hormonally active (2). Ectopic ACCs are extremely rare. This variant is believed to arise from cortical fragments left behind during embryologic migration and has been found close to the adrenal gland, along the path of gonadal descent, or in association with a gonad (2). The adrenal cortex is derived from the intermediate mesoderm in the region between the urogenital ridge and the root of the mesentery. The most frequent pelvic or groin location can be explained by the fact that the foetal adrenal cortex is often unencapsulated and develops in close contact with the gonads (3). The majority of ACCs are sporadic; however, ACC has been linked with several well-described genetic disease processes, including Li–Fraumeni syndrome, Beckwith–Wiedemann syndrome, and multiple endocrine neoplasia type 1 (MEN-1). We present the case of a 66-year-old female patient with previously undiagnosed MEN-1 syndrome in whom a functioning extra-adrenal ACC was diagnosed. To our knowledge, this is the first case report of extra-adrenal ACC in MEN-1 syndrome.

## Case presentation

A 66-year-old female was referred to our endocrinology clinic with a new diagnosis of diabetes. The patient had a history of parathyroidectomy for primary hyperparathyroidism in 2015, hypertension, and previous spinal decompressive surgery. She reported a 6-month history of significant weight gain and facial flushing. Examination revealed Cushingoid features. She had no family history of adrenal lesions or endocrinopathy.

## Investigation

Hormonal evaluation confirmed an ACTH-independent Cushing’s syndrome. Cortisol after 1 mg overnight dexamethasone suppression testing was 548 nmol/L (reference range (RR): < 50 nmol/L), with an undetectable ACTH <3 pg/mL (RR: 7.2–63.3 pg/mL). Midnight salivary cortisol was also considerably elevated at 11.2 nmol/L (RR: < 2.6 nmol/L). Dehydroepiandrosterone (DHEAS) was 5.3 μmol/L (RR: 0.5–5.6 umol/L) and androstenedione was 3.49 nmol/L (RR: 1.39–9.77 nmol/L), both of which were normal. Testosterone was suppressed <0.4 nmol/L (RR: 0.1–1.4 nmol/L).

Full blood count, renal profile, and liver profile were within normal limits. Recurrent hyperparathyroidism was noted with an elevated corrected calcium of 2.92 mmol/L (RR: 2.21–2.52 mmol/L) and a paired PTH of 71 pg/mL (RR: 15–65 pg/mL). Sestamibi and single-photon emission computed tomography (SPECT CT) findings were in keeping with a 0.8 cm right lower parathyroid adenoma deep within the mediastinum, close to the brachiocephalic arterial trunk and trachea.

**Figure 1:**
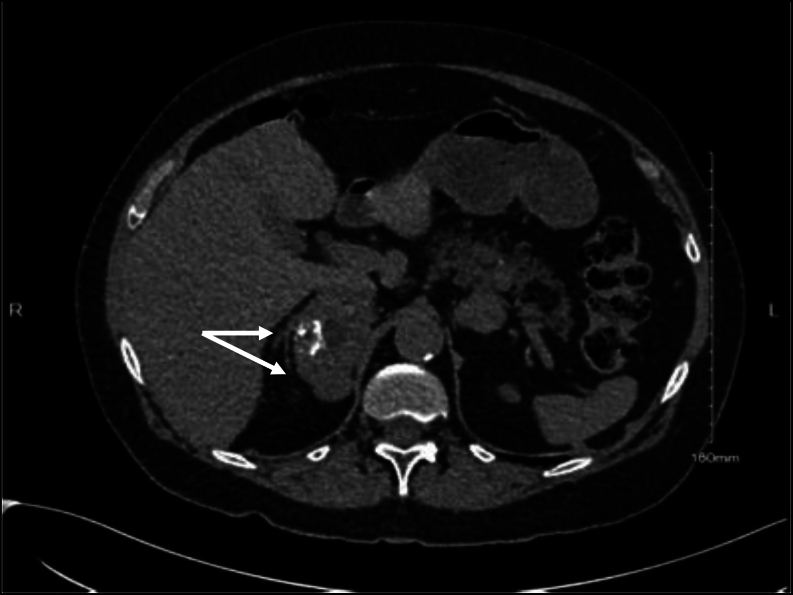
CT showing right-sided presumed adrenal lesion with internal calcification. Arrows indicate intact medial and lateral limb of the right adrenal gland with histology showing complete separation of ACC from the normal adrenal gland.

Adrenal CT was performed which demonstrated a 6 × 6 × 4.5 cm right-sided presumed adrenal lesion with Hounsfield units >20 (Fig. 1)*.* A distal body pancreatic lesion (2.5 × ‍1.6 cm) and bilateral pulmonary nodules (0.9 × 0.8 cm, 0.7 × 0.6 cm, 0.3 cm) were also noted. Both the right-sided presumed adrenal lesion and pancreatic lesion were fluorodeoxyglucose (FDG) avid on 18^F^ glucose PET CT. Octreotide scan and Gallium PET CT demonstrated increased tracer uptake in the distal pancreatic lesion but not in the lung lesions or adrenal lesion.

**Figure 2:**
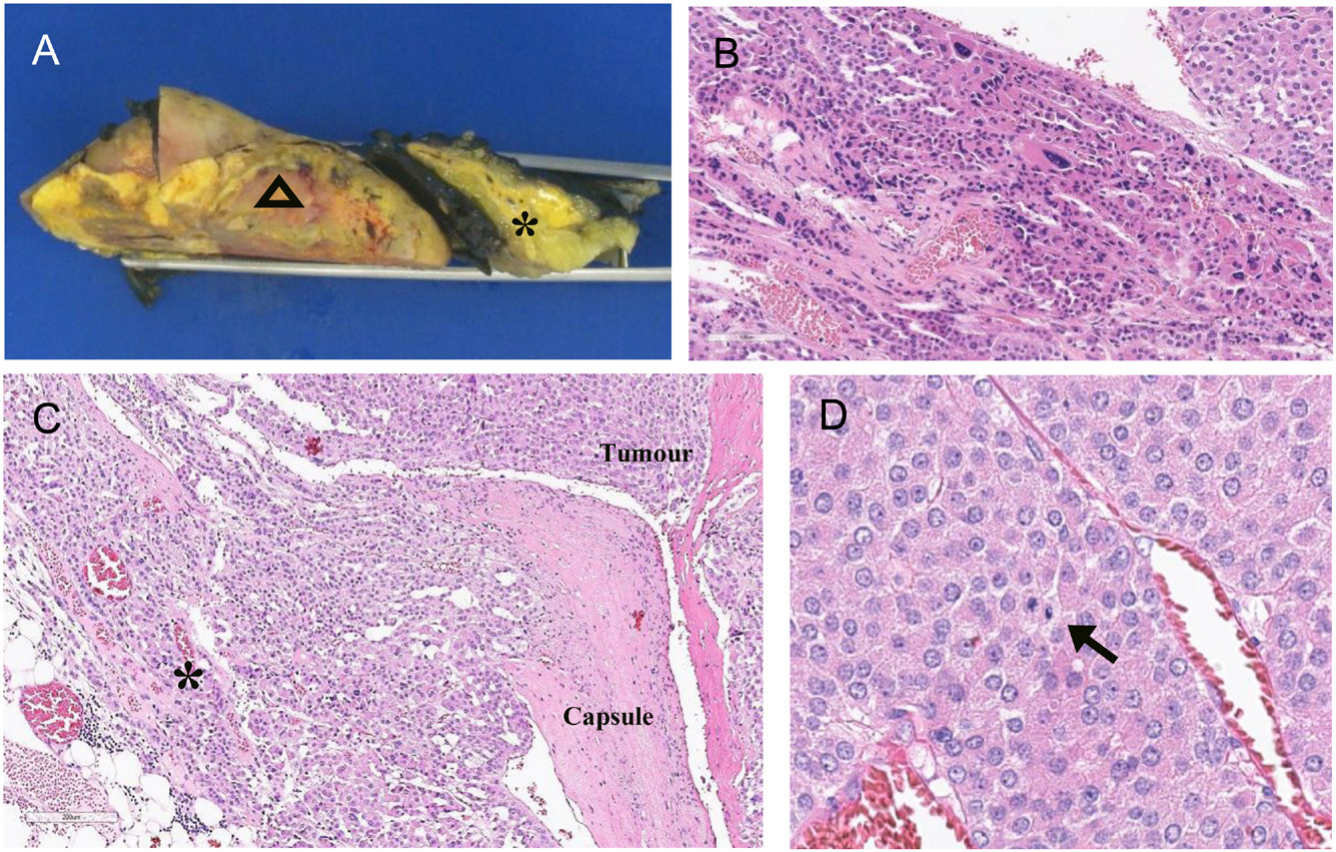
(A) Macroscopic image showing the adrenal cortical carcinoma (left) and the adjacent uninvolved adrenal gland (right). Triangle signifies adrenal cortical carcinoma. Asterisk signifies uninvolved adrenal gland. (B) Tumour showing epithelioid cells arranged in broad trabeculae with prominent nuclear pleomorphism (haematoxylin and eosin, original magnification 200×). (C) Tumour showing capsular invasion and extension into peri-tumoural fat (asterisk) (haematoxylin and eosin, original magnification 100×). (D) Tumour shows scattered mitotic figures (arrow, 4/50 HPF) with no atypical forms seen (haematoxylin and eosin, original magnification 200×).

She proceeded to have a right adrenalectomy. Histology (Fig. 2) was in keeping with a diagnosis of ACC arising from the peri-adrenal adipose tissue, an extra-adrenal cortical rest. Weiss score (Table 1) was 5/9 and Ki67 of 15%. The resected adrenal gland was normal. Right lower lobe lung biopsy confirmed metastatic ACC. Endoscopic ultrasound-guided biopsy of the pancreatic lesion was consistent with a well-differentiated neuroendocrine tumour, and biochemical testing revealed this to be an insulinoma. Genetic testing of leucocyte DNA was positive for MEN-1.

**Table 1:** The Weiss criteria used for distinguishing benign from malignant adrenal cortical tumours.

**Weiss criteria** **≥3 for adrenal cortical carcinoma**
Nuclear grade III or IV (Fuhrman) >5 mitoses/50 HPF* Atypical mitotic figures* Clear or vacuolated cells ≤25% of tumour Diffuse architecture (> 33% of tumour) Necrosis Venous invasion* (of smooth muscle walled vessels) Sinusoidal invasion Capsular invasion

*major criteria; 1 is required to diagnose adrenal cortical carcinoma (14)

## Treatment

Mitotane therapy was commenced for the management of metastatic ACC with a target range of 14–20 mg/L. The pancreatic neuroendocrine tumour remains under surveillance with input from a neuroendocrine tumour multidisciplinary team and medical therapy in the form of diazoxide, with good biochemical control. Cinacalcet was prescribed to manage hypercalcaemia in the setting of recurrent hyperparathyroidism.

## Outcome and follow-up

Most recent follow-up CT TAP, 2 years following diagnosis, reveals an unremarkable surgical bed. The lung nodules remain sub-centimetre in size, and the pancreatic lesion is unchanged. This patient is tolerating mitotane therapy well, with her most recent mitotane level to target at 19.6 mg/L on Mitotane 1.5 g three times a day. She continues on replacement hydrocortisone and fludrocortisone. Her calcium is stable, most recently at 2.69 mmol (2.21–2.52 mmol/L). She is asymptomatic and continues on cinacalcet 30 mg once daily and diazoxide 25 mg twice daily.

## Discussion

In this report, we describe a female patient with previously undiagnosed MEN-1 syndrome in whom a functioning extra-adrenal ACC was diagnosed.

This patient presented to our clinic with a new diagnosis of diabetes and a clinical history and examination suggestive of Cushing’s syndrome. ACC generally presents in one of three ways: symptoms of hormone excess, non-specific abdominal symptoms, or are diagnosed incidentally on imaging. Approximately half of ACC lesions are hormonally active. The most common syndrome of hormone excess is Cushing’s syndrome, which occurs in approximately 45% of cases. A mixed picture of Cushing’s syndrome and virilisation due to cortisol and androgen hypersecretion is seen in 20–30% of cases. Patients with non-functional ACC can present with non-specific symptoms such as abdominal pain, weight loss, or poor appetite. Studies report that 20–30% of patients with ACC are asymptomatic and incidentally diagnosed (1, 2).

Although tumours of the adrenal gland are common, ACC is rare. A minority of cases occur in the setting of genetic disease. Initial insights into the genes and signaling pathways involved in ACC tumourigenesis came from studies of these genetic diseases, including Li–Fraumeni syndrome, Lynch syndrome, familial adenomatous polyposis, and MEN-1 (1). ACC is a recognised core tumour in Li–Fraumeni syndrome because of TP53-germline mutations. Overall, 50% to 80% of all children diagnosed with ACC have an underlying diagnosis of Li–Fraumeni syndrome. The frequency of TP53-germline mutations in ACC decreases with age to less than 10% in adulthood. Particularly in ACC patients, TP53-germline mutations are diverse, either due to low penetrance alleles or de novo mutations. Given the relatively high prevalence of TP53-germline mutations in patients with ACC and the high frequency of de novo mutations, genetic testing for TP53 mutations should be recommended for all, even in the absence of a positive family history (4, 5). The prevalence of ACC in Lynch syndrome and familial adenomatous polyposis is higher than observed in the general population. Lynch syndrome is caused by germline mutations in DNA mismatch repair genes MLH1, MSH2, MSH6, and PMS2. In a prospective study of 94 patients with ACC, the prevalence of Lynch syndrome was 3.2%. This is comparable to the prevalence of Lynch syndrome in colorectal cancer (2–4%) and endometrial cancer (1–5%), supporting that ACC is a Lynch syndrome-associated malignancy (6). Patients with familial adenomatous polyposis have increased adrenocortical adenomas compared to the general population. However, the absolute risk for ACC is much lower compared to colorectal cancers in these patients and no dedicated screening is recommended (7).

MEN-1 is an autosomal dominant disease resulting from a mutation in the MEN-1 tumour suppressor gene on chromosome 11q13. It is classically and most frequently characterised by the development of tumours in tissues of endocrine origin such as parathyroid glands, pituitary gland, and neuroendocrine pancreas. Involvement of the adrenal gland has been reported in approximately 40% of patients, most commonly bilateral hyperplasia or adenomas. The majority of patients with MEN-1 with adrenal lesions, including the patient described in this report, have cortical involvement combined with a history of pancreatic neuroendocrine tumours (8). ACCs are reported in approximately 2.5–6% of MEN-1 patients and are considered a rare manifestation of the syndrome (9). However, the malignant potential of the adrenal lesion in MEN-1 syndrome is striking. In several cases of MEN-1, accelerated growth of prior known adrenal lesions and subsequent diagnosis of ACC is described. This suggests that ACC may arise from precursor lesions in the setting of MEN-1 (3, 8, 9). Other studies suggest that every adrenal enlargement in MEN-1 may progress to highly aggressive ACC. Newly diagnosed adrenal lesions in MEN-1 should be followed closely with short-interval imaging, and a lower threshold for surgical removal is suggested (9).

The patient in this report subsequently was found to have a family history of MEN-1 syndrome and known primary hyperparathyroidism at presentation. Parathyroid tumours, resulting in primary hyperparathyroidism, are the most common feature of MEN-1 and occur in 95% of MEN-1 patients. Primary hyperparathyroidism is often the earliest laboratory or clinical manifestation. Furthermore, it is estimated that 1–18% of patients diagnosed with primary hyperparathyroidism will have MEN-1 (10). This case highlights the importance of recognising patients at risk of MEN-1 and arranging prompt genetic testing. Early identification allows for appropriate screening to be initiated and close clinical follow-up.

The ACC in this report is extra-adrenal, and the right adrenal gland was morphologically normal. Ectopic adrenal tissue is frequently found in newborns and children; however, it usually atrophies during infancy (3). Ectopic ACCs are extremely rare. They are believed to arise from cortical fragments arrested during embryologic migration and have been found close to the adrenal gland or along the path of gonadal descent (2). They have also been reported in the liver (11) and abdominal wall (12). A case of retroperitoneal ACC was described in a gentleman with Lynch syndrome (13). To our knowledge, this is the first case report of extra-adrenal ACC in MEN-1 syndrome.

In conclusion, adrenal lesions in MEN-1 syndrome have significant malignant potential. This case highlights the importance of screening for MEN-1 in at-risk patients and the need for close clinical follow-up.

## Declaration of interest

The authors declare that there is no conflict of interest that could be perceived as prejudicing the impartiality of the research reported.

## Funding

This research did not receive any specific grant from any funding agency in the public, commercial, or not-for-profit sector.

## Patient consent

Written informed consent for the publication of the clinical details and clinical images was obtained from the patient.

## Author contribution statement

D Green – Leading role: conceptualisation, data curation, writing. K Richards – Supporting role: data curation. B Doyle – Supporting role: data curation, supervision. C Thompson – Supporting role: supervision. A Hill – Supporting role: supervision. M O’Reilly – Supporting role: supervision. M Sherlock – Leading role: conceptualisation, editing, supervision.
